# Composition and genetics of malaria vector populations in the Central African Republic

**DOI:** 10.1186/s12936-016-1431-2

**Published:** 2016-07-26

**Authors:** Mamadou Ousmane Ndiath, Karin Eiglmeier, Marina Lidwine Olé Sangba, Inge Holm, Mirdad Kazanji, Kenneth D. Vernick

**Affiliations:** 1 G4 Malaria Group, Institut Pasteur of Bangui, BP 923, Bangui, Central African Republic; 2 G4 Malaria Group, Institut Pasteur of Madagascar, BP 1274, Ambohitrakely, 101 Antananarivo, Madagascar; 3 Unit of Insect Vector Genetics and Genomics, Department of Parasites and Insect Vectors, Institut Pasteur, 28 rue du Docteur Roux, 75015 Paris, France; 4 Unit of Hosts, Vectors and Pathogens (URA3012), CNRS, 28 rue du Docteur Roux, 75015 Paris, France; 5 Faculté des Sciences et Techniques, Université d’Abomey Calavi, Cotonou, Benin; 6 Virology Department, Institut Pasteur de Bangui, Bangui, Central African Republic; 7 Institut Pasteur de la Guyane, 23 Avenue Pasteur, BP 6010, 97306 Cayenne Cedex, French-Guiana

**Keywords:** *Anopheles*, Insecticide resistance, Malaria, Bangui, Central African Republic

## Abstract

**Background:**

In many African countries malaria has declined sharply due to a synergy of actions marked by the introduction of vector control strategies, but the disease remains a leading cause of morbidity and mortality in Central African Republic (CAR). An entomological study was initiated with the aim to characterize the malaria vectors in Bangui, the capital of CAR, and determine their vector competence.

**Methods:**

A cross-sectional entomological study was conducted in 15 sites of the district of Bangui, the capital of CAR, in September–October 2013 and a second collection was done in four of those sites between November and December 2013. Mosquitoes were collected by human landing catch (HLC) indoors and outdoors and by pyrethrum spray catch of indoor-resting mosquitoes. Mosquitoes were analysed for species and multiple other attributes, including the presence of *Plasmodium falciparum* circumsporozoite protein or DNA, blood meal source, 2La inversion karyotype, and the L1014F *kdr* insecticide resistance mutation.

**Results:**

Overall, 1292 anophelines were analysed, revealing a predominance of *Anopheles gambiae* and *Anopheles funestus*, with a small fraction of *Anopheles coluzzii*. Molecular typing of the *An. gambiae* complex species showed that *An. gambiae* was predominant (95.7 %) as compared to *An*. *coluzzii* (2.1 %), and *Anopheles arabiensis* was not present. In some areas the involvement of secondary vectors, such as *Anopheles coustani,* expands the risk of infection. By HLC sampling, *An. funestus* displayed a stronger endophilic preference than mosquitoes from the *An. gambiae* sister taxa, with a mean indoor-capture rate of 54.3 % and 67.58 % for *An. gambiae* sister taxa and *An. funestus,* respectively. Human biting rates were measured overall for each of the species with 28 or 29 bites/person/night, respectively. Both vectors displayed a strong human feeding preference as determined by blood meal source, which was not different between the different sampling sites. *An. coustani* appears to be highly exophilic, with 92 % of HLC samples captured outdoors. The mean CSP rate in head-thorax sections of all *Anopheles* was 5.09 %, and was higher in *An. gambiaes.l.* (7.4 %) than in *An. funestus* (3.3 %). CSP-positive *An. coustani* were also detected in outdoor HLC samples. In the mosquitoes of the *An. gambiae* sister taxa the *kdr*-*w* mutant allele was nearly fixed, with 92.3 % resistant homozygotes, and no susceptible homozygotes detected.

**Conclusions:**

This study collected data on anopheline populations in CAR, behaviour of vectors and transmission levels. Further studies should investigate the biting behaviour and susceptibility status of the anophelines to different insecticides to allow the establishment of appropriate vector control based on practical entomological knowledge.

**Electronic supplementary material:**

The online version of this article (doi:10.1186/s12936-016-1431-2) contains supplementary material, which is available to authorized users.

## Background

Despite national and international efforts, the burden of malaria morbidity remains high, especially in tropical regions of Africa [1]. In Central African Republic (CAR), malaria remains a serious public health problem and is the leading cause of death among children. In the major part of the country, malaria is hyperendemic but the only detailed information available comes from the city of Bangui, in particular from the Hôpital Communautaire [2–4]. Only insufficient and inconsistent data are available from other areas of CAR due to the lack of resources and violent political conflicts. The country is plagued by shortages of essential drugs and logistical constraints, and access of the population to health care is far from being a reality.

Since July 2006, the Global Fund Programme for Malaria, through the Global Fund to Fight AIDS, Tuberculosis and Malaria has distributed free insecticide-treated nets (ITNs) to pregnant women and children under 5 years in all health centres and vaccination sites in CAR, but malaria remains endemic throughout the country. Today, preventing malaria relies on treatment of patients, and mosquito control interventions [5, 6] with the latter primarily based on the use of ITNs and indoor residual spraying. However, these efforts are threatened by the emergence of insecticide resistance in insect vectors [7].

Vector control can be effective in the long term only through a thorough understanding of the transmission cycles, the biology and ecology of vectors [8, 9]. Many malaria transmitting mosquitoes belong to species complexes with behavioural, environmental and genetic differences that influence their role as vectors [8, 10] and they can show important differences in malaria transmission [11, 12].

The knowledge about mosquito populations and malaria vectors in CAR is very limited. The most recent study focused on the epidemiology of yellow fever [13]. To the best of the authors’ knowledge, no study has been conducted to determine the precise epidemiological role of mosquito species in malaria transmission and to collect a reference data set of entomological information that can serve to define appropriate control strategies adapted to the status of the species concerned.

The current study provides an inventory of malaria vectors present in Bangui, the capital of CAR, comprising data on vector biology, vector behaviour, the distribution of the L1014F *kdr* resistance mutation and importance of anopheline species in transmission, constituting a baseline for entomologic and epidemiologic interventions.

## Methods

### Study area

CAR is a vast, sparsely populated country, covering 623,000 km^2^ with a population in 2015 of about 4.9 million inhabitants [14]. The country is surrounded by Cameroon to the West, Chad to the North, Sudan and Southern Sudan to the East, the Democratic Republic of Congo and the Congo to the South. The CAR climate is subequatorial, with temperatures varying from 19 to 32 °C and a rainy period between April and November. Malaria transmission occurs during the entire year, with peaks at the beginning and the end of the rainy season.

## Study design

All mosquitoes in this study were collected during two cross-sectional studies. The first collection was performed during September–October 2013 (in the middle of the rainy season) and was conducted in 15 districts of Bangui, the capital of CAR: Gobongo, Gbanikola, Malimaka, Gbaya–Dombia, Greboutou, Taoka Saint Paul, PK 10, Yamangala, Yakité Dado, Ile de singe, Lakouanga, Dendégué II, Galabadja, Saïdou, Cité Jean XXIII; (Additional files 1, 2). The second collection covered four districts (Gbanikola, Taoka Saint Paul, PK 10, Ile de singe) and was done in November–December. This second collection, initially planned as a broader longitudinal study of 2 consecutive years, had to be interrupted after the first weeks of collection because of political violence in December 2013. In all districts, GPS coordinates of mosquito collection sites were recorded.

### Mosquito collections

Adult mosquitoes were collected by human landing catch (HLC) and pyrethrum spray catch (PSC). Hourly HLC were made on adult volunteers from 18:00 to 6:00 h for two consecutive nights at two indoor and two outdoor sites in typical households of the central part of the village.

Pyrethrum spray catches were conducted in five rooms in houses where insecticides or repellents had not been used during the previous week and that were different from houses used for HLC. For PSC, deltamethrin (Yotox^®^) was sprayed inside the closed rooms for 30–45 s. After 10 min, dead or immobilized mosquitoes fallen on white sheets were collected in Petri dishes and brought to the laboratory.

### Mosquito identification by morphology, blood-meal determination and CSP detection

Anophelines were identified by the morphological identification keys of Gillies and De Meillon [15]. The blood meal of blood-fed females captured by PSC was squashed onto Whatman No. 1 filter paper and tested by ELISA to distinguish between bovine, ovine, caprine (sheep and goat), equine (horse and donkey) or chicken origin, as previously described [16]. The presence of circumsporozoite protein (CSP) of *Plasmodium falciparum* was determined using an enzyme-linked immunosorbent assay (ELISA-CSP) performed on the dissected head and thorax section [17].

### Mosquito DNA isolation

Genomic DNA was extracted from individual mosquitoes of the second collection, regardless of whether they were visibly blood fed or not, using DNAzol according to the manufacturer’s recommendations (Invitrogen, CA, USA). The total genomic DNA from each mosquito was resuspended in 100 µl H_2_O and stored at −20 °C until use.

### Species determination and genotyping

All DNA samples from the second collection were typed for species, molecular form and 2La inversion karyotype as described previously [18]. Briefly, species status and molecular form were determined by either or both of two diagnostic assays, the SINE200 X6.1 assay [19] or the molecular assay described by Fanello et al. [20], to allowed the discrimination of *Anopheles arabiensis, Anopheles gambiae* (formerly S molecular form) and *Anopheles coluzzii* (formerly M molecular form). If these assays failed to give diagnostic fragments, the ribosomal gene ITS2 spacer region or a region of the mitochondrial *cytochrome C oxidase I* gene were PCR amplified and analysed. The 2La inversion type was determined using the published molecular assay [21]. Oligonucleotide primers with a 5′ fluorescent label were used in these three different assays that are based on polymerase chain reactions (PCR), permitting the sizing of the generated PCR fragments using an ABI Genetic Analyzer 3730 and called using Genemapper 3.5 (Applied Biosystems, CA, USA) for result analysis as described [18].

### Typing of the *kdr*-*w* L1014F insecticide resistance mutation

Typing of the West African *kdr*-*w* mutation (L1014F), thereafter identified as *kdr*-*w,* was done as described [18], briefly using the following primers and PCR conditions: Agd1 5′-ATA GAT TCC CCG ACC ATG-3′, Agd2 5′-AGA CAA GGA TGA TGA ACC-3′, Agd3 5′-AAT TTG CATT ACT TAC GAC A-3′, Agd4 5′-CTG TAG TGA TAG GAA ATT TA-3′. PCR conditions consisted of an initial denaturing step at 94 °C for 5 min followed by 30 cycles of 94 °C for 30 s, 55 °C for 30 s, 72 °C for 15 s and a final extension of 72 °C for 5 min. The Agd1 and Agd4 primers were labeled with a fluorophore and the PCR fragments were separated on an ABI Genetic Analyzer 3730. All samples displayed a control band of 293 bp while resistant (1014 phe-coding) individuals displayed an additional 196 bp band and susceptible (1014 leu-coding) individuals a 136 bp band whereas heterozygous mosquitoes showed all three bands.

### Detection of *Plasmodium* parasites by PCR

*Plasmodium* parasite presence was ascertained by PCR amplification of a portion of the *Plasmodium* mitochondrial *cytochrome b* gene (*cytB*) following modifications of the protocols from Fornadel et al. [22] and Hasan et al. [23] as described in [24]. Amplicons were visualized by agarose gel electrophoresis. This *Plasmodium* detection assay was performed on DNA samples originating from all individual mosquitoes of the second collection, independent of whether the mosquito was visibly blood fed or not.

### ITS2 analysis

The rDNA nuclear internal transcribed spacer 2 (ITS2) was amplified by PCR using primers described by Beebe and Saul [25], essentially according to the recommendations of the MR4 protocol [26]. In anopheline species the length of the amplified ITS2 fragments can vary according to the species. The amplicons were visualized by agarose gel electrophoresis and representatives of PCR fragments of different sizes were sequenced in the forward and reverse direction. Sequencing was performed at a commercial laboratory according to the recommended protocol for BigDye terminator cycle sequencing (Applied Biosystems, USA). Sequences were then compared by BLASTN against public databases to determine mosquito species from the most significant alignment.

### Amplification of mosquito mitochondrial *cytochrome C oxidase I* gene

A 749 bp region of the mitochondrial cytochrome C oxidase subunit 1 gene (*COI*) gene was amplified by PCR using the primers COI-forward 5′-GGA GGA TTT GGA AAT TGA TTA GTT CC-3 and COI-reverse 5′-GCT AAT CAT CTA AAA ATT TTA ATT CC-3′, essentially as published [26]. The amplicons were sequenced in the forward and reverse direction by a commercial laboratory according to the recommended protocol for BigDye Terminator Cycle Sequencing (Applied Biosystems, USA). Consensus sequences were generated from forward and reverse reads and compared with the Barcode of Life [27] database for species identification.

### Data analyses

Qualitative data were compared using Pearson Chi Square or Fisher exact test and statistical analyses were performed with Stata 10.1. A *p* value of 0.05 or less was considered as significant. The human biting rate was estimated from the number of bites per person per night sampled by HLC. The indoor-capture rate was calculated as the proportion of the number of mosquitoes captured indoors among the total number of mosquitoes captured indoors and outdoors by HLC. The anthropophily rate was calculated as the proportion of freshly fed mosquitoes that had taken at the time of capture an exclusively human blood meal among all fed mosquitoes. The infection rate was calculated as the proportion of *Plasmodium* positive mosquitoes to the total number of malaria vectors.

## Results

In total, 1292 anophelines were collected between September and December 2013. The first collection, over 60 person-nights in 15 districts of Bangui, led to the capture of 825 anophelines by HLC (Additional files 1, 2) and 227 anophelines by PSC. In the second collection, 197 anophelines were captured by HLC and 43 by PSC. Besides the predominant *An. gambiae* sister taxa and *Anopheles funestus*, other *Anopheles* species such as *An. coustani, Anopheles natalensis* and *Anopheles pharoensis* were also captured (Table 1).

**Table 1:** Summary of the anophelines collected in Bangui by HLC and by PSC

Species	Human landing catch		Pyrethrum spray catch	
	Indoor	Outdoor	Indoor	Total
*An. gambiae* sister taxa	328	276	198	802
*An. funestus*	171	82	69	322
*An. coustani*	7	81	1	89
*An. natalensis*	13	55	0	68
*An. pharoensis*	0	9	2	11
Total	519	503	270	1292

Mosquitoes were captured during the first collection (September–October 2013) in 15 districts of Bangui and during the second collection (November–December 2013) in four districts

### Human biting rate and nocturnal biting cycle

**Fig. 1:**
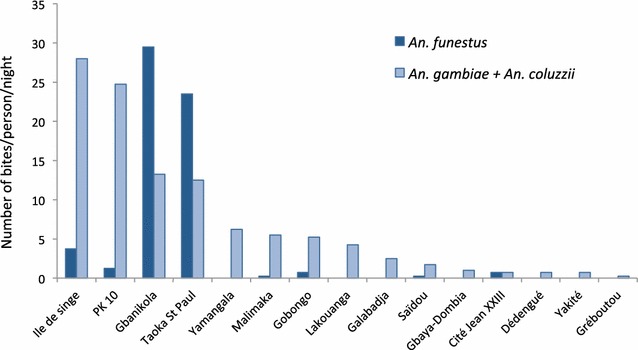
Number of bites per person per night of *Anopheles funestus* and mosquitoes belonging to the *Anopheles gambiae* sister taxa (September–October 2013). Mosquitoes (n = 825) were collected by HLC in 15 districts of Bangui

The human biting rate (HBR) variations of mosquitoes of the *An. gambiae* sister taxa and *An. funestus* collected in 15 districts of Bangui are shown in Fig. 1. *An. gambiae* sister taxa are present in all districts with a maximum aggressiveness observed in Ile de singe, with 28 bites/person/night (B/P/N) and PK10 (24 B/P/N) whereas *An. funestus* is more prevalent in Gbanikola (29.5 B/P/N) and Taoka St. Paul (23.5 B/P/N). In the four districts these major malaria vectors are sympatric, showing a high HBR and, therefore, resulting in the highest combined bite density per night amongst the districts in this survey.

**Fig. 2:**
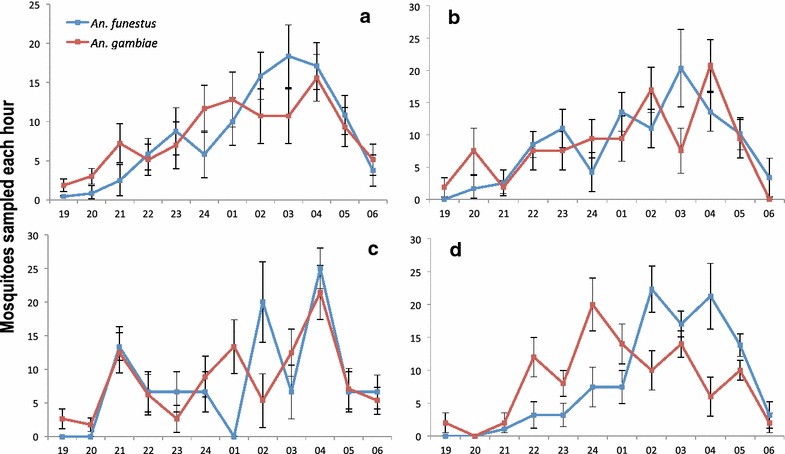
Hourly aggressiveness of *Anopheles funestus* and *Anopheles gambiae* sister taxa populations (n = 825) collected by HLC in September–October 2013 (% and 95 % confidence intervals). **a** Global result from the 15 districts, **b** from Gbanikola, **c** from Ile de singe and **d** from Taoka St. Paul

Night-biting cycles were similar for *An. gambiae* sister taxa mosquitoes and *An. funestus*. Mosquito aggressiveness gradually increased after 19:00 h until about 03:00 h, and then sharply declined (Fig. 2a). A similar trend was observed at sites where the higher number of sampled mosquitoes permitted a graphical representation, notably at Gbanikola (Fig. 2b) and Ile de singe (Fig. 2c). At Taoka St. Paul, although sample sizes per time point were smaller, the difference in aggressiveness between the mosquito species of this sample set seemed to be more pronounced. There mosquitoes from the *An. gambiae* sister taxa were more aggressive during the period from 21:00–23:00 h, while *An. funestus* biting was more active from about 02:00–05:00 h (Fig. 2d).

### Indoor-capture rate and human blood index

Of the total 1022 specimens sampled by HLC in both collections, 519 (50.78 %) were collected indoor and 503 (49.21 %) outdoor. The mean indoor-capture rate was 54.3 % and 67.58 % for *An. gambiae* sister taxa and *An. funestus,* respectively. There was a significant difference in indoor-capture rate between *An. gambiae* sister taxa and *An. funestus* (Chi Square = 12.9, df = 1, *p* < 0.0005) with *An*. *funestus* appearing more endophilic while *An*. *gambiae* sister taxa were captured in both locations. By contrast, the mean outdoor-capture rate of *An*. *coustani* was 92.0 %.

**Fig. 3:**
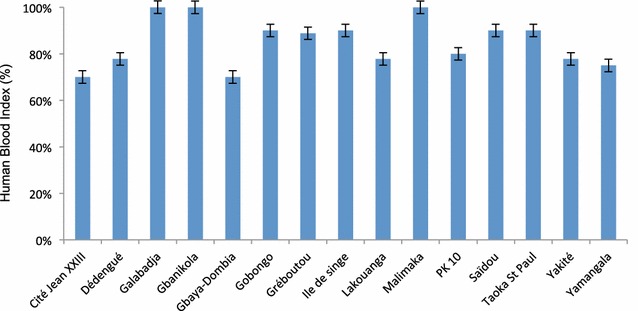
Human blood index (proportion and 95 % confidence interval) in 149 mosquitoes from *Anopheles gambiae* sister taxa (September–October 2013). Mosquitoes were collected by PSC in 15 districts of Bangui

Blood meal origin was determined in 149 *An*. *gambiae* sister taxa and 21 *An*. *funestus* sampled by PSC (n = 170). Of these, 133 were strictly of human origin (73.23 %) and 31 were from animals (18.23 %), with 24 of dog origin (77.42 %), 7 from bovine (22.58 %) and 6 consisted of a mixture of human and animal blood (3.53 %). The human blood index (HBI) of *An. gambiae* sister taxa mosquitoes and *An. funestus* was (81.8 %) and (52.3 %) respectively, indicating a high degree of human-vector contact (Additional file 3). However, no significant difference of HBI was observed in these two important vector species (Chi Square = 1.31, *p* = 0.251). No significant variation by district was observed in the anthropophilic rate of *An. gambiae* sister taxa mosquitoes (Chi Square = 1.50, *p* = 1.00) (Fig. 3).

### Determination of *Plasmodium falciparum* circumsporozoite protein rate

The *P. falciparum* circumsporozoite protein (CSP) rate was determined by ELISA-CSP. Of the 825 *Anopheles* specimens of the first collection captured by HLC and tested by ELISA-CSP, 42 mosquitoes were positive for the CSP antigen (Table 2), resulting in a mean CSP of 5.09 % with 7.4 % and 3.33 % for *An. gambiae* sister taxa and *An. funestus,* respectively (Chi Square = 4.15, *p* = 0.041). This was also reflected in the results from the *An*. *gambiae* sister taxa where the infection rate in indoor-captured mosquitoes of 10.3 % (n = 233, number of positive mosquitoes = 24) that was significantly higher than those of outdoor-captured mosquitoes with 4.06 % (n = 197, number of positive mosquitoes = 8, Additional file 4) (Chi Square = 5.2, *p* = 0.02). In addition, two *An*. *coustani* (captured outdoors) were positive for the CSP antigen in the district Ile de singe. This species presents a very strong tendency to exophily and may constitute a real risk of malaria transmission. None of the *An. natalensis* mosquitoes was found positive for CSP. Thus, *P. falciparum* positive mosquitoes were found at all collection sites except at Dédengué, Gbaya–Doumbia and Greboutou, where the number of captured mosquitoes was very low (Table 2).

**Table 2:** Detection of CSP antigen in mosquitoes collected by HLC in the 15 districts of Bangui (September–October 2013)

District	*An. funestus*		*An. gambiae* sister taxa		*An. coustani*		*An. natalensis*		Total	
	Tested	CSP+	Tested	CSP+	Tested	CSP+	Tested	CSP+	Tested	CSP+
Cité Jean XIII	3	1	3	0	0	NA	0	NA	6	1
Dédengué	0	NA	3	0	0	NA	0	NA	3	0
Galabadjia	0	NA	10	1	0	NA	0	NA	10	1
Gbanikola	118	4	53	6	3	0	15	0	189	10
Gbaya-Doumbia	0	NA	4	0	0	NA	5	0	9	0
Gobongo	3	0	21	2	0	NA	0	NA	24	2
Greboutou	0	NA	1	0	0	NA	7	0	8	0
Ile de Singe	15	1	112	3	82	2	13	0	222	6
Lakouanga	0	NA	17	1	0	NA	5	0	22	1
Malimaka	1	0	22	1	0	NA	0	NA	23	1
PK10	5	0	99	6	2	0	7	0	113	6
Saïdou	1	0	7	1	0	NA	0	NA	8	1
Taoka St Paul	94	2	50	6	0	NA	16	0	160	8
Yakité	0	NA	3	1	0	NA	0	NA	3	1
Yamangala	0	NA	25	4	0	NA	0	NA	25	4
Total	240	8	430	32	87	2	68	0	825	42
Mean CSP rate	3.33 %		7.4 %		2.29 %		0 %		5.09 %	

Mosquitoes were captured during the first collection (September–October 2013) in 15 districts of Bangui and during the second collection (November–December 2013) in four districts

### Mosquito identification and chromosomal characterization

Of the 243 anophelines captured in the second collection (November–December 2013), 183 have been identified as mosquitoes belonging to the *An. gambiae* sister taxa using the molecular diagnostic assays with a predominance of *An. gambiae* (n = 175) and few *An. coluzzii* (n = 4). For four samples their species could not be determined but they were identified as being part of the *An. gambiae* sister taxa. No *An. arabiensis* mosquitoes were present in this collection. The 60 specimens that could not be identified with these assays were characterized using the nuclear internal transcribed spacer 2 (ITS2) based diagnostics [28]. In anophelines the length of the amplified ITS2 fragments varies according to the species and representative amplicons of three different sizes were obtained from the tested samples and sequenced. Sequence comparison by BLASTN against public databases identified the approximately 750 bp fragment as originating from *An. funestus* (n = 44) and an approximately 650 bp fragment led to the identification of *An. coustani* (n = 2) mosquitoes. Fragments of 600 bp were characteristic for mosquitoes belonging to the *An. gambiae* sister taxa.

To determine the species of those mosquitoes that could not be identified with the ITS2-based assay (n = 14), a region of the mitochondrial *cytochrome oxidase subunit I* (*COI*) gene was amplified by PCR and sequenced. The sequences of the amplicons were compared to the Barcode of Life database and permitted the identification of *An. pharoensis* (n = 11) and also confirmed the former identification of the *An. coustani* mosquitoes. Three samples could not be typed, probably due to poor DNA quality. *COI* based species identification has gained increasing importance due to the universal primers that amplify the gene in many species. The degree of variations found in the sequenced amplicons permits the distinction of many animals and has been shown to be useful for mosquito species identification [29].

Analysis of the paracentric 2La inversion performed on 183 specimen of the *An*. *gambiae* sister taxa showed that 37.7 % presented the karyotype 2L +/ 2La (n = 69), 39.3 % 2L +/ 2L + (n = 72) and 21.3 % 2La/2La (n = 39). Three samples did not give results.

The detection of the *kdr* insecticide resistance allele L1014F frequency (*kdr*-*w* type) in 183 mosquitoes of the *An*. *gambiae* sister taxa revealed a high prevalence of this resistance conferring variant present in the voltage-gated sodium channel encoding *para* gene [30]. Thus 92.3 % of the *An*. *gambiae* sister taxa mosquitoes presented a homozygous resistant profile of the type RR (n = 169), 4.4 % a heterozygous profile RS (n = 8). Six samples did not give results. None of the tested *An*. *gambiae* sister taxa mosquitoes was found to be homozygous for the insecticide sensitive wild type *kdr* allele L1014.

**Fig. 4:**
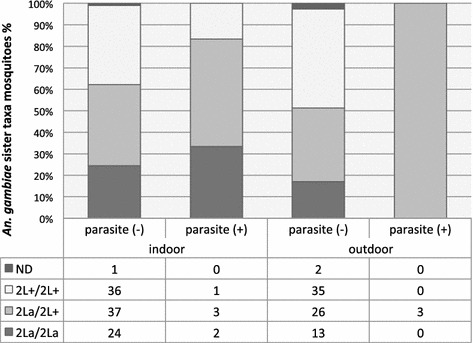
Relation between 2L chromosomal forms, *Plasmodium* parasite detection and place of capture (November–December 2013). *Anopheles* *gambiae* sister taxa (n = 183) were collected indoors and outdoors by HLC and PSC in four districts of Bangui

Detection of *P. falciparum* parasites in the 183 mosquitoes of the *An*. *gambiae* sister taxa by PCR [22–24] revealed 9 parasite positive mosquitoes, one was captured by PSC and 8 by HLC (infection rate = 4.91 %). In terms of indoor-captured (n = 104) and outdoor-captured mosquitoes (n = 79), this rate corresponded to 5.77 % (n = 6) and 3.79 % (n = 3) (Chi square = 0.33, *p* = 0.56) respectively (Fig. 4). All molecular attribute data for mosquitoes of the second collection (November–December 2013) is available (Additional file 5: Table S1).

## Discussion

This study provides for the first time in at least 50 years a characterization of potential malaria vectors present in the CAR, and a first evaluation of their status of insecticide resistance considering the presence of the *kdr* insecticide resistance allele L1014F. These results showed that in Bangui the most efficient malaria vectors, *An. coluzzii*, *An. gambiae* and *An*. *funestus,* are sympatric and play an important role in malaria transmission. They appear well adapted to humans and their environment, being highly anthropophilic. In addition, secondary vector species such as *An. coustani* were also detected and could play a significant role in transmission. Similar *Anopheles* diversity was also reported from Cameroon [2], Gabon [31], Chad [32] and in the Democratic Republic of Congo [33].

In this study, data about anopheline aggressiveness showed an important activity of mosquitoes of the *An. gambiae* sister taxa and *An. funestus* during the early night hours (particularly after midnight), which generally corresponds to the period when people are in bed. Evaluation of the ITN coverage showed that in 2014 66.8 % of the population regularly used bed nets during the night, however by observation during the current surveys many of these bed nets were in poor physical condition, therefore representing an inefficient barrier for blood-seeking mosquitoes.

*Anopheles* densities were high in certain districts and local variations in the number and species of captured anophelines by HLC were detected. Similar observations have been made in Senegal [34], Benin [35] and Cameroon [36]. *Anopheles gambiae* sister taxa are known to exhibit opportunistic feeding behaviour and therefore blood meal identification is important in understanding vectorial capacity of malaria vectors and transmission dynamics [37]. In this study, mosquitoes of the *An. gambiae* sister taxa were highly anthropophilic, even though most of the households kept cattle near their houses and no difference of anthropophilic biting rates was observed according to the district. In addition, a high level of infection rate was observed, which was greater in mosquitoes captured indoors than outdoors.

The genomic studies, done with the mosquitoes of the second collection, showed the predominance of *An. gambiae* (n = 175), with very little presence of *An. coluzzii* (n = 4). Other studies also demonstrated the predominance of *An. gambiae* in this part of Central Africa. *An. gambiae* have a larval development that usually requires high rainfall in contrast to the *An. coluzzii*, generally more adapted to drier conditions with a larval development very similar to *An. arabiensis* [11, 38, 39].

In the two sample sets *Plasmodium* parasites were detected by either ELISA-CSP (in the first collection) or by PCR (in the second collection) and the differences in the infection rates displayed higher parasite prevalence in mosquitoes of the *An. gambiae* sister taxa in the first collection. This difference is probably not due to a sensitivity problem. *Plasmodium* detection by PCR, done on DNA prepared from individual mosquitoes, was shown to be more sensitive than ELISA-CSP [40] and could even have detected parasites present in a freshly taken blood meal whereas ELISA-CSP, done on dissected head-thorax fractions, detects established *Plasmodium* infections.

The study revealed a high prevalence of the *kdr*-*w* L1014F mutated allele in mosquitoes of the *An. gambiae* sister taxa in Bangui, similar to the observed rates reported in other countries [30, 41–43]. Several studies have suggested that the use of agricultural pesticides favored the emergence of *kdr* mutations and facilitated the spread of the insecticide resistance associated allele within mosquito populations [44]. Although this point was not specifically investigated, it is possible that changes in agricultural practice in certain district of Bangui have contributed to the emergence of this resistance. On the other hand, the exposure to the insecticides of ITNs may also be a factor responsible for the *kdr* allele diffusion [35, 45]. Additional studies would be needed to investigate the origin of *kdr* allele diffusion and their impact on *Anopheles* insecticide susceptibility.

In CAR, the WHO malaria control gold standards were not met [1]. Due to political instability, the use of anti-malarial drugs and of ITNs by the population was not high and most malaria attacks were not treated. In many countries, the choice of the anti-malarial drug (artemisinin-based combinations) used for the first-line treatment and the universal deployment of ITNs were the most important factors responsible for reduction of malaria infection and malaria morbidity [46]. The current situation in Bangui brings to mind the status observed in different countries before vector control intervention [34, 47, 48]. In these countries the introduction of untreated bed nets as first vector control interventions were reported to shift the biting hours, resulting in an increase of the exophilic tendency [7, 42, 49]. However, the absence of structured vector control strategies, such as ITNs of good quality, is probably one of the origins of this high anthropophilic rate observed in Bangui. Therefore, considerable efforts will be needed to reduce the level of man-vector contact to decrease malaria transmission as it has been observed in other countries [47, 50].

## Conclusion

The knowledge of malaria vector biology is a prerequisite for the design and implementation of current and future vector control strategies. Control methods to be put in place have to take into account the different vector populations but also the possibility that these various mosquito species or populations could transmit different *Plasmodium* genotypes carrying their resistance alleles. CAR is in a difficult political situation and faces a malaria epidemiological situation that is almost catastrophic. Thus, this preliminary study raises important points for further investigations and decisions.

## Additional files

10.1186/s12936-016-1431-2 Predominant anopheline species captured in 15 districts of Bangui by Human Landing Catch (September–October 2013).
10.1186/s12936-016-1431-2 Number of *Anopheles* collected by Human Landing Catch in 15 districts of Bangui (September–October 2013).
10.1186/s12936-016-1431-2 Anophelines collected by Pyrethrum Spray Catch (PSC) in 15 districts of Bangui (September–October 2013). Blood-fed females were identified and blood meal origin was determined by enzyme-linked immunosorbent assay (ELISA).
10.1186/s12936-016-1431-2 All *Anopheles* mosquitoes collected indoors and outdoors by Human Landing Catch in 15 districts of Bangui (September–October 2013) were tested by ELISA-CSP to determine their *Plasmodium* infection status and to calculate their circumsporozoite rates.
10.1186/s12936-016-1431-2 Molecular attribute data for mosquitoes collected in 4 districts of Bangui (November–December 2013).

## Abbreviations

CAR: Central African Republic
HLC: human landing catch
PSC: pyrethrum spray catch
CSP: circumsporozoite protein
ITN: insecticide-treated net
bp: base pairs
CI: confidence interval
