# Supplementary material for: Composition and genetics of malaria vector populations in the Central African Republic
Source: Malar J. 2016 Jul 26;15:387. doi: 10.1186/s12936-016-1431-2 (PMC4960874; doi:10.1186/s12936-016-1431-2)

Additional File 1: Predominant anopheline species captured in 15 districts of Bangui by Human Landing Catch (September-October 2013)

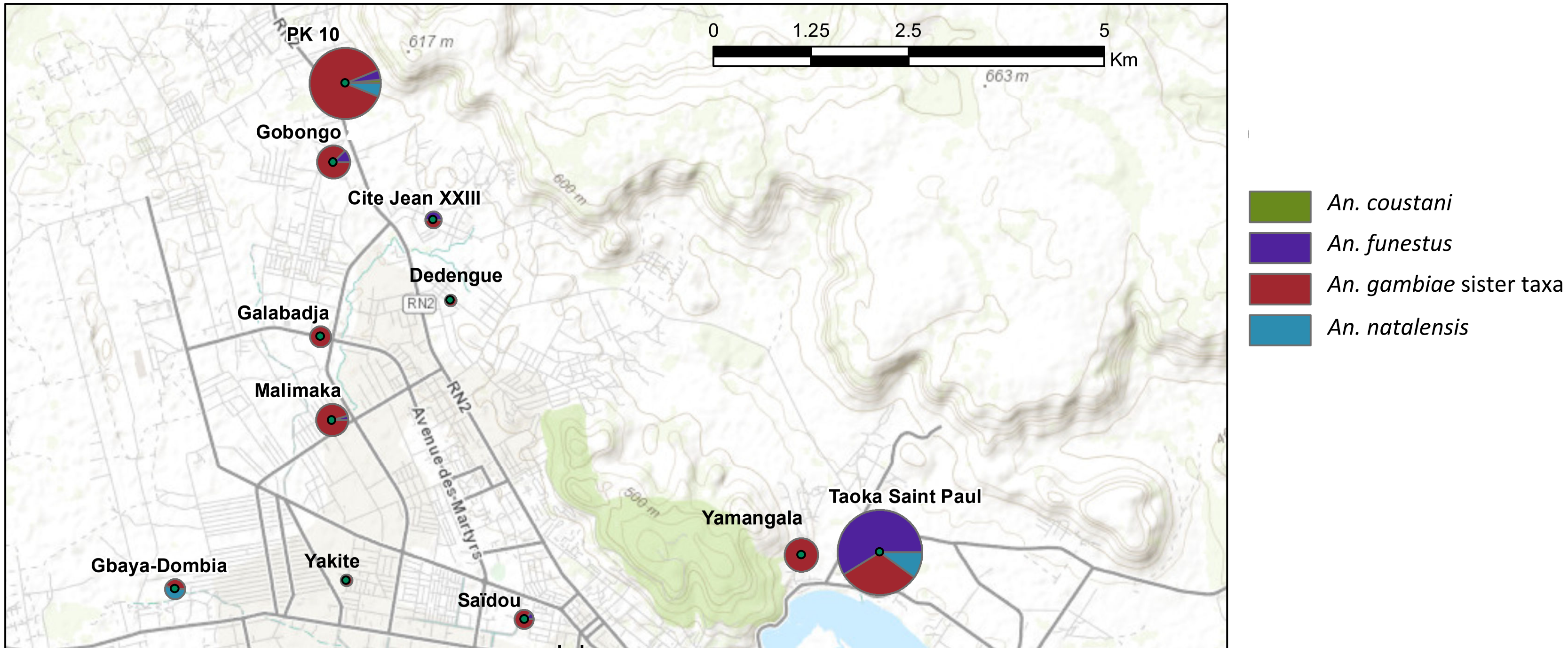

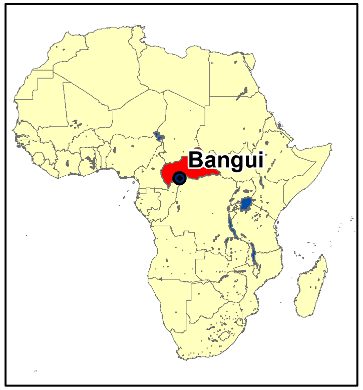

Supplement: Supplementary file 1 — 10.1186/s12936-016-1431-2 Predominant anopheline species captured in 15 districts of Bangui by Human Landing Catch (September–October 2013). [file 12936_2016_1431_MOESM1_ESM.pdf]
