# Supplementary material for: Composition and genetics of malaria vector populations in the Central African Republic
Source: Malar J. 2016 Jul 26;15:387. doi: 10.1186/s12936-016-1431-2 (PMC4960874; doi:10.1186/s12936-016-1431-2)
Supplement: Supplementary file 2 — 10.1186/s12936-016-1431-2 Number of Anopheles collected by Human Landing Catch in 15 districts of Bangui (September–October 2013). [file 12936_2016_1431_MOESM2_ESM.pdf]

	An. gambiae sister taxa		*An. funestus*		*An. coustani*		*An. natalensis*	
**Sites**	**Indoor**	**Outdoor**	**Indoor**	**Outdoor**	**Indoor**	**Outdoor**	**Indoor**	**Outdoor**
Cite Jean XXIII	2	1	1	2	0	0	0	0
Dedengue	3	0	0	0	0	0	0	0
Galabadja	4	6	0	0	0	0	0	0
Gbanikola	29	24	86	32	0	3	2	13
Gbaya-Dombia	1	3	0	0	0	0	0	5
Gobongo	7	14	0	3	0	0	0	0
Greboutou	1	0	0	0	0	0	1	6
Ile de singe	78	34	10	5	6	76	4	9
Lakouanga	9	8	0	0	0	0	1	4
Malimaka	7	15	0	1	0	0	0	0
PK 10	54	45	4	1	1	1	3	4
Saïdou	1	6	0	1	0	0	0	0
Taoka St Paul	23	27	62	32	0	0	2	14
Yakite	0	3	0	0	0	0	0	0
Yamangala	14	11	0	0	0	0	0	0
**Total**	**233**	**197**	**163**	**77**	**7**	**80**	**13**	**55**
	**430**		**240**		**87**		**68**	

<u>Additional File 2</u>: Number of *Anopheles* collected by Human Landing Catch in 15 districts of Bangui (September-October 2013).
