# Supplementary material for: Composition and genetics of malaria vector populations in the Central African Republic
Source: Malar J. 2016 Jul 26;15:387. doi: 10.1186/s12936-016-1431-2 (PMC4960874; doi:10.1186/s12936-016-1431-2)
Supplement: Supplementary file 3 — 10.1186/s12936-016-1431-2 Anophelines collected by Pyrethrum Spray Catch (PSC) in 15 districts of Bangui (September–October 2013). Blood-fed females were identified and blood meal origin was determined by enzyme-linked immunosorbent assay (ELISA). [file 12936_2016_1431_MOESM3_ESM.pdf]

	Number of collected mosquitoes (n=229)		Number of blood-fed mosquitoes (n=193)		Number of mosquitoes tested for blood meal origin (n=170)		Blood meal source in *An. gambiae* sister taxa (n=149)			Blood meal source in *An. funestus* (n=21)		
**Sites**	***An. gambiae* sister taxa**	***An. funestus***	***An. gambiae* sister taxa**	***An. funestus***	***An. gambiae* sister taxa**	***An. funestus***	**Human**	**Animal**	**Mixture**	**Human**	**Animal**	**Mixture**
Cite Jean XXIII	12	0	11	0	10	0	7	3	0	0	0	0
Dedengue	14	2	12	1	10	1	7	2	1	0	1	0
Galabadja	13	2	11	1	10	1	9	0	1	0	1	0
Gbanikola	15	6	12	4	10	4	10	0	0	3	1	0
Gbaya-dombia	12	3	11	2	10	2	7	3	0	1	1	0
Gobongo	13	2	11	1	10	1	9	1	0	0	1	0
Greboutou	13	3	12	2	10	2	8	1	1	2	0	0
Ile de singe	13	4	12	2	10	2	9	1	0	1	1	0
Lakouanga	13	1	11	0	10	0	7	2	1	0	0	0
Malimaka	12	2	11	1	10	1	10	0	0	1	0	0
PK 10	14	4	12	3	10	3	8	2	0	1	2	0
Saïdou	12	2	12	1	10	1	9	1	0	0	1	0
Taoka St Paul	14	4	12	2	10	2	9	1	0	2	0	0
Yakite	11	1	11	1	10	1	7	2	1	0	1	0
Yamangala	10	2	9	2	9	0	6	2	1	0	0	0
**Total**	**191**	**38**	**170**	**23**	**149**	**21**	**122**	**21**	**6**	**11**	**10**	**0**
**Human blood index (%)**							**81.8%**			**52.3%**		

Additional File 3: Anophelines collected by Pyrethrum Spray Catch (PSC) in 15 districts of Bangui (September-November 2013). Blood-fed females were identified and blood meal origin was determined by enzyme-linked immunosorbent assay (ELISA).
