# Supplementary material for: Composition and genetics of malaria vector populations in the Central African Republic
Source: Malar J. 2016 Jul 26;15:387. doi: 10.1186/s12936-016-1431-2 (PMC4960874; doi:10.1186/s12936-016-1431-2)
Supplement: Supplementary file 4 — 10.1186/s12936-016-1431-2 All Anopheles mosquitoes collected indoors and outdoors by Human Landing Catch in 15 districts of Bangui (September–October 2013) were tested by ELISA-CSP to determine their Plasmodium infection status and to calculate their circumsporozoite rates. [file 12936_2016_1431_MOESM4_ESM.pdf]

	Human Landing Catch		Number of tested mosquitoes		Number of positive mosquitoes by CSP-ELISA		Circumsporozoite rate (%)	
	Indoor	Outdoor	Indoor	Outdoor	Indoor	Outdoor	Indoor	Outdoor
An. gambiae sister taxa	233	197	233	197	24	8	10.3%	4.06%
An. funestus	163	77	163	77	6	2	3.68%	2.59%
An. coustani	7	80	7	80	0	2	0	2.5%
An. natalensis	13	55	13	55	0	0	0	0
**Total**	**416**	**409**	**416**	**409**	**30**	**12**	**7.21%**	**2.93%**

Additional File 4: All *Anopheles* mosquitoes collected indoors and outdoors by Human Landing Catch in 15 districts of Bangui (September-November 2013) were tested by ELISA-CSP to determine their *Plasmodium* infection status and to calculate their circumsporozoite rates.
